# Development of a Triplex TaqMan Real-Time PCR Assay for Simultaneous Detection of Duck Hepatitis B Virus, Duck Adenovirus Type 3, and *Streptococcus gallolyticus* subsp. *pasteurianus*

**DOI:** 10.3390/vetsci13070692

**Published:** 2026-07-15

**Authors:** Mingfa Yang, Wei Yu, Xiaofang Chen, Wei Zhang, Qingwen Meng, Changwen Li, Jiasen Liu, Changyou Xia

**Affiliations:** 1 Heilongjiang Provincial Key Laboratory of Laboratory Animal and Comparative Medicine, State Key Laboratory for Animal Disease Control and Prevention, Harbin Veterinary Research Institute, Chinese Academy of Agricultural Sciences, Harbin 150069, China; yangmingfa@caas.cn (M.Y.); chenxiaofang202606@163.com (X.C.); mengqingwen@caas.cn (Q.M.); lichangwen@caas.cn (C.L.); 2 National Poultry Laboratory Animal Resource Center, Harbin Veterinary Research Institute, Chinese Academy of Agricultural Sciences, Harbin 150069, China; yuwei1919@126.com (W.Y.); zhangwei@caas.cn (W.Z.)

**Keywords:** duck hepatitis B virus, duck adenovirus 3, *Streptococcus gallolyticus* subsp. *Pasteurianus*, triplex real-time quantitative PCR

## Abstract

Duck hepatitis B virus (DHBV), Duck adenovirus 3 (DAdV-3) and *Streptococcus gallolyticus* subsp. *pasteurianus* (SGSP) are three distinct pathogens associated with infectious diseases in waterfowl. Clinical differentiation among these agents is often difficult due to overlapping signs, underscoring the need for accurate, rapid, and simultaneous diagnostic tools to support early detection and effective disease surveillance. In this study, we developed and validated a triplex TaqMan real-time quantitative PCR (qPCR) assay for the simultaneous detection and quantification of DHBV, DAdV-3, and SGSP. The assay indicated high analytical sensitivity, achieving detection limits of 10 copies/µL for each target, with all standard curves showing excellent linearity (R^2^ = 0.999). The evaluation of specificity showed that the triplex qPCR assay had high specificity for the three target pathogens, as it did not yield any cross-reactive signals with other pathogens examined. Intra-assay and inter-assay coefficients of variation for recombinant plasmid standards were consistently <2%, indicating high reproducibility. Meanwhile, the assay was employed to investigate the epidemiology of the three pathogens in ducks. The positive rates for DHBV, DAdV-3 and SGSP were 73.95% (159/215), 11.16% (24/215) and 37.21% (80/215), respectively. The co-infection rates were 5.58% (12/215) for DHBV and DAdV-3, 27.91% (60/215) for DHBV and SGSP, 2.33% (5/215) for DAdV-3 and SGSP, and 2.33% (5/215) for all three pathogens. In summary, this triplex qPCR assay displays excellent specificity, sensitivity and repeatability, which may serve as a reliable testing tool for routine clinical diagnosis, epidemiological monitoring and co-infection screening of the three target pathogens in locally sampled duck populations.

## Simple Summary

Duck farming contributes significantly to the global livestock industry and economic development. However, infections caused by duck hepatitis B virus (DHBV), duck adenovirus type 3 (DAdV-3), and *Streptococcus gallolyticus* subsp. *pasteurianus* (SGSP) often lead to severe liver damage, high mortality, and frequent co-infections in ducks, resulting in substantial economic losses. These pathogens usually cause similar clinical symptoms and pathological lesions, making rapid and accurate on-site differential diagnosis difficult. In this study, a triplex TaqMan real-time PCR method was established for the simultaneous detection of DHBV, DAdV-3, and SGSP in a single reaction tube. The method exhibited high specificity, sensitivity, and repeatability, with a limit of detection of 10 copies/μL for each pathogen. A total of 215 clinical duck samples were tested, and the results showed good concordance between this triplex qPCR assay and individual conventional PCR assays. Overall, this triplex qPCR method offers a rapid and reliable strategy for simultaneous detection and differential identification of DHBV, DAdV-3, and SGSP, and could be applicable for clinical diagnosis and epidemiological surveillance of the three pathogens within the sampled duck population.

## 1. Introduction

The primary pathogens causing hepatocyte necrosis in ducks include Duck hepatitis B virus (DHBV), Duck adenovirus type 3 (DAdV-3) and *Streptococcus gallolyticus* subsp. *pasteurianus* (SGSP). Co-infection with these pathogens is common in ducks and poses a serious threat. Notably, these pathogens can induce symptoms such as hepatocyte necrosis, myocardial injury, pericardial effusion, and hemorrhage in ducks. These clinical manifestations are often indistinguishable. Co-infections involving DHBV, DAdV-3 and SGSP may exacerbate disease severity, potentially elevating mortality and causing substantial economic losses to the duck farming industry.

The duck hepatitis B virus (DHBV) belongs to the genus *Avihepadnavirus* in the family *Hepadnaviridae* [1,2]. The DHBV genome is a covalently closed circular partially double-stranded DNA (cccDNA), consisting of a complete long strand (negative strand) and an incomplete short strand (positive strand), approximately 3.0 kb in length [3,4,5]. DHBV is harmful to the liver of the naturally infected ducklings, causing hepatic hemorrhage and hepatocyte dysfunction. Compared with adult ducks, duck embryos and young ducklings are far more susceptible to DHBV infection; vertical transmission dominates its transmission pathway, which aligns with the infection pattern of human HBV [6,7]. Furthermore, an epidemiological report indicates that DHBV frequently co-infects with other pathogens in waterfowl [8].

Duck adenovirus 3 (DAdV-3) is a newly identified DAdV serotype that has been discovered in recent years. The agent exhibits high pathogenicity in ducks, with macroscopic pathological changes predominantly manifested as enlargement, hemorrhage and necrotic lesions in both the liver and kidney of infected individuals [9]. As a member of adenoviruses, DAdV-3 possesses canonical adenoviral traits, including spherical non-enveloped viral particles, icosahedral symmetry, and a linear double-stranded DNA genome of 43,842 bp in length [10]. The morbidity of the disease ranges from 40% to 55%, with a mortality rate of 35–43%, posing a great threat to duck farms in China [11]. Previous studies have reported that co-infections with DAdV-3 and other pathogens are detected frequently in poultry farms [12].

*S. gallolyticus* subsp. *pasteurianus*, *S. gallolyticus* subsp. *gallolyticus*, and *S. gallolyticus* subsp. macedonicus form a single DNA cluster belonging to the same *S. gallolyticus genospecies* [13,14]. SGSP is being increasingly recognized as a cause of meningitis and bacteremia in newborns [15]. It causes neonatal sepsis and splenic abscess in humans, as well as acute septicemia in goslings and turkey poults [16]. In a previous study, the highly virulent strain causing meningitis and splenic necrosis was isolated from the brains of ducklings [17].

Numerous conventional PCR and real-time PCR assays have been reported for individual detection of DHBV, DAdV-3 or SGSP. However, each platform only identifies one pathogen per reaction and cannot distinguish co-infections in a single tube. Furthermore, existing multiplex detection panels for waterfowl pathogens are limited to either multiple viral species or multiple bacterial strains within one single taxonomic group; few available assays enable concurrent screening covering avian hepadnavirus, adenovirus and streptococcus simultaneously [18,19,20]. Conventional viral isolation and serological detection methods involve cumbersome protocols, long turnaround times, substantial labor input, and suboptimal analytical sensitivity and specificity. To overcome the above technical limitations, species-specific primer and probe sets were designed for the DHBV S gene, DAdV-3 fiber-2 gene, and SGSP 16S rRNA gene in this research. We developed a triplex TaqMan real-time quantitative PCR assay to simultaneously detect DHBV, DAdV-3, and SGSP, which could serve as an alternative molecular approach to assist the surveillance and clinical control of duck hepatic infectious diseases.

## 2. Materials and Methods

### 2.1. Pathogenic Nucleic Acids and Clinical Samples

The genomes (DNA or RNA) of DHBV, DAdV-3, SGSP, Duck plague virus (DPV), *Escherichia coli* (*E.coli*), *Pathogenic Salmonella* (*Salm.*), *Riemerella anatipestifer* (*RA*), Muscovy duck parvovirus (MDPV), Duck tembusu virus (DTMUV), Duck hepatitis A virus type 1 (DHAV-1), *Salmonella enterica* (*S. enterica*), *Pasteurella multocida* (*P. multocida*), *Streptococcus gallolyticus* subsp. *Gallolyticus* (SGSG), and *Streptococcus gallolyticus* subsp. *Macedonicus* (SGSM) were stored in the State Key Laboratory for Animal Disease Control and Prevention, Harbin Veterinary Research Institute, Chinese Academy of Agricultural Sciences. Duck liver samples (*n* = 215) obtained from numerous duck farms across Heilongjiang Province were graciously supplied by the Laboratory of Harbin Veterinary Research Institute, Chinese Academy of Agricultural Sciences. All 215 clinical liver samples were collected during routine epidemiological surveillance of duck farms. Samples were derived from naturally infected ducks aged 7–45 days with typical liver injury clinical signs, such as lethargy, reduced feed consumption and hepatic hemorrhage. Post-mortem liver parenchyma tissues were aseptically harvested following standard sampling procedures, placed in sterile cryogenic tubes and temporarily preserved at −20 °C on the farm before long-term storage at −80 °C in the laboratory prior to nucleic acid extraction. Notably, no extra invasive treatments or harmful manipulations were administered to the experimental animals throughout this research. Based on the experimental design of this project, the Institutional Review Board of Harbin Veterinary Research Institute has granted an exemption, confirming that formal ethical review and approval procedures are not mandatory for this study.

### 2.2. Nucleic Acid Extraction

We isolated total DNA from duck liver tissue using the VAMNE Magnetic Pathogen DNA/RNA Kit (Vazyme, Nanjing Vazyme Biotech Co., Ltd., Nanjing, China) per the manufacturer’s Instructions. The extracted DNA was dissolved in 85 µL of nuclease-free water and stored at −80 °C for subsequent analyses.

Commercially available universal tissue nucleic acid extraction kits based on silica-coated magnetic beads are widely validated for co-extraction of viral and bacterial DNA. The magnetic particles are wrapped with silica dioxide on the outer layer, which adopts the classic silica–nucleic acid adsorption principle consistent with silica spin columns. The silica surface shows no bias toward DNA templates from different pathogens, and no differential loss of viral or bacterial nucleic acids occurs during purification. This unified extraction workflow ensures consistent recovery of both viral and bacterial templates, which is critical for reliable detection of mixed viral–bacterial infections via the triplex qPCR assay.

### 2.3. Primers and Probe Design

To ensure optimal detection performance, the S gene of DHBV, the *fiber-2* gene of DAdV-3 and the 16S rRNA specific region gene of SGSP were aligned using SnapGene 6.0.2 software (GSL Biotech LLC, Chicago, IL, USA) to identify highly conserved regions. The 16S rRNA gene was chosen as the target of SGSP to enhance qPCR sensitivity for trace clinical samples. This gene contains subspecies-specific variable regions that support the discrimination of closely related *S. gallolyticus* subspecies. Through multi-sequence alignment of SGSP, SGSM and SGG 16S rRNA sequences, the primer–probe set was designed to cover SGSP-unique SNP sites. Terminal mismatches inhibit non-specific amplification of near-related subspecies, and BLAST (https://blast.ncbi.nlm.nih.gov/, accessed on 10 March 2026) analysis verified that the oligonucleotides only perfectly match SGSP templates, ensuring the assay exclusively detects SGSP without cross–reactivity.

Three primer pairs and corresponding TaqMan probes were designed to target the conserved regions of DHBV (GenBank accession no. MF471769), DAdV-3 (GenBank accession no. KR135164), and SGSP (GenBank accession no. JN798591). Primers and probes were designed using Primer Express 3.0.1 software (Applied Biosystems, Foster City, CA, USA). Their specificity was subsequently confirmed by BLAST analysis against the NCBI nucleotide database. To enable simultaneous triplex target detection, each probe was labeled with distinct 5′ reporter fluorophores (FAM, VIC, Cy5) and matched 3′ quencher moieties (BHQ1, BHQ1, MGB), respectively. The detailed information of the primers and probes is shown in Table 1. All primers and probes were synthesized by RuiBiotech, Beijing, China.

### 2.4. Preparation of Standard Plasmid

**Figure 1:**
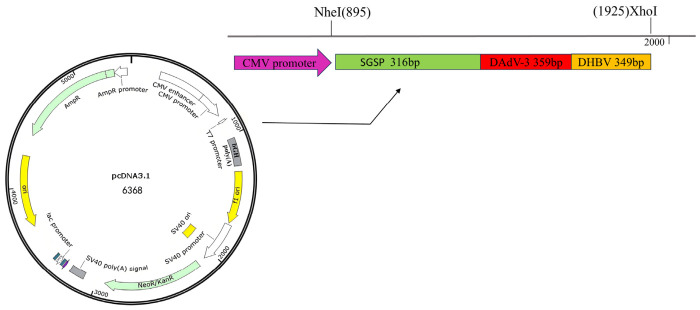
Standard plasmid Fusion-Std-SGSP-DAdV3-DHBV containing three conserved gene fragments: SGSP (316 bp), DAdV-3 (359 bp), and DHBV (349 bp). The full-length sequences of each segment are provided separately in Table S1.

To reduce assay variability and experimental costs, a fusion standard plasmid containing partial gene sequences of the three pathogens was constructed instead of three individual plasmids. A DNA fragment containing partial sequences of the SGSP 16S rRNA specific region, DAdV-3, and DHBV was synthesized by RuiBiotech Co., Ltd. (Beijing, China). This fragment was subsequently inserted into the pcDNA3.1(+) cloning vector, generating a recombinant standard plasmid designated Fusion-Std-SGSP-DAdV3-DHBV, which was used for further analysis (Figure 1). The respective full sequence of each segment is listed in three separate rows of Supplementary Table S1. Its concentration was measured using a Thermo NanoDrop Lite apparatus (Thermo Fisher Scientific, Waltham, MA, USA) and was converted into copy numbers via the following formula: copies/µL = (A260 (ng/µL) × 10^−9^ × 6.02 × 10^23^)/(DNA length × 650). The standard plasmids were serially diluted 10-fold to a concentration gradient of 1 × 10^8^ to 1 × 10^0^ copies/μL with EASY Dilution (TaKaRa, Dalian, China).

### 2.5. Establishment and Optimization of the Triplex qPCR System

To optimize the reaction parameters of the triplex qPCR, including primer and probe concentrations as well as annealing temperature, a series of conditions were tested. The reaction system volume was 20 µL:10 µL of 2× Premix Ex Taq (TaKaRa, Dalian, China), 2 µL of standard plasmid, three mixed pairs of primers and three probes of different volumes, and sterile distilled water to a final volume of 20 µL. All amplification reactions were carried out using an ABI QuantStudio 5 real-time PCR instrument (Thermo Fisher Scientific, Waltham, MA, USA) with the following program: 95 °C for 5 s, followed by 40 cycles of 95 °C for 10 s and 56 °C for 30 s. To obtain an optimized reaction system for the triplex qPCR, we screened multiple addition volumes of 10 µM primer and probe working solutions. The primer volumes ranged from 0.3 µL to 0.9 µL, and probe volumes ranged from 0.3 µL to 0.7 µL. An amount of 1 × 10^7^ copies/µL of the standard plasmid was used as a template for optimization. Gradient annealing temperatures ranging from 54 °C to 60 °C (54 °C, 56 °C, 58 °C, 60 °C) were evaluated to optimize the amplification efficiency of the triplex qPCR system. Corresponding fluorescence intensity and Ct values were compared to determine the optimal thermal cycling parameters.

### 2.6. Establishment of Standard Curves for the Triplex qPCR

Using the final reaction conditions, the plasmids were quantified and 10-fold serially diluted to a final concentration of 1 × 10^0^ copy/μL. Plasmids with a series of copy numbers ranging from 1 × 10^8^ to 1 × 10^2^ copies/μL were used as templates for the triplex qPCR assay. The standard curve was established by plotting the logarithm of the standard copy number against the corresponding Ct value.

### 2.7. Specificity of the Triplex qPCR

To evaluate the specificity of the triplex qPCR assay, the genomic DNA of DHBV, DAdV-3, SGSP, DPV, MDPV, *E. coli*, *Salm*, *RA*, *S. enterica*, *P. multocida*, SGSG and SGSM, the cDNA of DHAV-1, and DTMUV were tested using the optimized reaction system. DHBV, DAdV-3 and SGSP nucleic acids were used as positive controls, and nuclease-free water was used as the negative control.

### 2.8. Sensitivity of the Triplex qPCR

To determine the sensitivity of the triplex qPCR assay, 10-fold serial dilutions of the standard plasmid ranging from 1 × 10^8^ to 1 × 10^0^ copies/µL were prepared in 10× Tris-EDTA buffer (pH 7.4) to construct a standard curve and determine the limit of detection (LOD) of the assay. Serial dilutions of fusion standard plasmids (100, 10, 1 copy/µL) were subjected to triplex TaqMan qPCR to determine the LOD. Three independent experimental runs were performed, with 20 technical replicates set for each concentration per run (60 total replicates per concentration). Consistent with MIQE guidelines, the LOD of each target pathogen was defined as the lowest plasmid concentration achieving a positive detection rate ≥ 95% across all replicates [24]. Two-sided 95% Wilson score confidence intervals were calculated for positive detection rates to assess statistical uncertainty of the detection probability at each concentration gradient. Samples exhibiting an exponential fluorescent amplification curve that crossed the preset threshold before cycle 36 (Ct value < 36) were classified as positive detections. Based on this judgment criterion, the positive detection ratios of standard plasmids at three concentration gradients (100, 10, and 1 copy/µL) were determined.

### 2.9. Repeatability Evaluation of the Triplex qPCR

To assess intra- and inter-assay coefficients of variation (CV) for Ct values acquired via the triplex qPCR platform, we generated 10-fold serial dilutions of the fusion standard plasmid with concentrations ranging from 1 × 10^3^ to 1 × 10^7^ copies/µL.

### 2.10. Detection of Clinical Samples by the Triplex qPCR

All DNA samples were tested in triplicate using the optimized triplex qPCR assay. To assess the diagnostic performance of the established assay, the same clinical samples were subjected to parallel testing via conventional PCR: Conventional PCR for DAdV-3 detection was performed in accordance with Fujian Provincial Local Standard DB35/T 1872-2019 [21]. Notably, there are no national or industrial standards available for the detection of DHBV, DAdV-3 and SGSP in China, and only DAdV-3 has a regional testing standard issued by Fujian Province. Therefore, we used the standardized PCR protocol from this provincial standard as the reference method to compare the performance of the established triplex qPCR assay. DHBV was detected using the PCR method described by Wang et al. (2013) [22]; and SGSP was detected using the PCR method described by Wilson et al. (1990) [23]. The reaction system and protocol for DAdV-3 (DB35/T 1872-2019) [21], DHBV (Wang et al., 2013) [22], and SGSP (Wilson et al., 1990) [23] are detailed in Table S2, respectively.

## 3. Results

### 3.1. Optimization of Triplex qPCR

**Figure 2:**
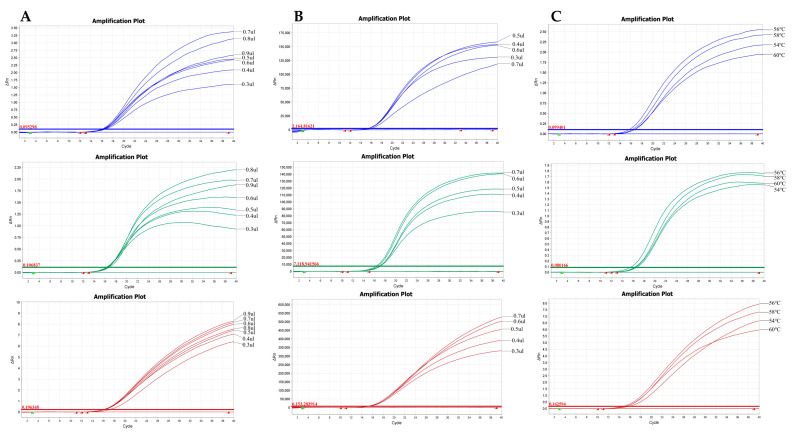
Optimization of the triplex qPCR method. (**A**) Primer volume optimization: optimal forward/reverse primer volumes were 0.7 µL (DAdV-3), 0.8 µL (SGSP), and 0.9 µL (DHBV). (**B**) Probe volume optimization: optimal probe volumes were 0.5 µL (DAdV-3), 0.6 µL (SGSP), and 0.7 µL (DHBV). (**C**) Annealing temperature optimization of the amplification protocol. The optimal annealing temperature was 56 °C.

After optimization, the optimal reaction parameters, including annealing temperature and primer/probe concentrations, were determined. The triplex qPCR reaction mixture was established as follows: the primers volumes were 0.7 µL for DAdV-3, 0.8 µL for SGSP, and 0.9 µL for DHBV; the probes volumes were 0.5 µL for DAdV-3, 0.6 µL for SGSP, and 0.7 µL for DHBV (Figure 2). The finalized reaction system is detailed in Table 2. A temperature of 56 °C was identified as the optimal annealing temperature, which yielded the highest amplification efficiency (Figure 2). The amplification curves, maximum ΔRn, and average Ct values obtained at different annealing temperatures are shown in Figure S1 and Table S3.

### 3.2. Establishment of the Standard Curve

**Figure 3:**
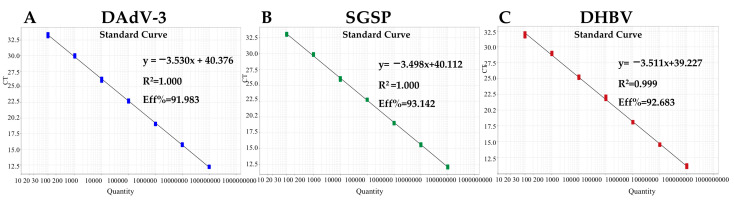
Standard curve for the triplex qPCR method. (**A**) Standard curve for DAdV-3; (**B**) Standard curve for SGSP; (**C**) Standard curve for DHBV.

A 10-fold serial dilution was performed on the standard plasmid, and seven dilutions, 1 × 10^8^ to 1 × 10^2^ copies/μL, were selected as templates to construct the standard curve for the triplex qPCR assay. Figure 3 shows the correlation coefficients (R^2^), equation slopes and amplification efficiencies (E = (10^−(1/slope)^ − 1) × 100) for each target: DAdV-3, 1.000, −3.530, and 91.983%; SGSP, 1.000, −3.498, and 93.142%; and DHBV, 0.999, −3.511, and 92.683%. A strong linear correlation was observed between initial template concentrations and Ct values.

### 3.3. Specificity Analysis

**Figure 4:**
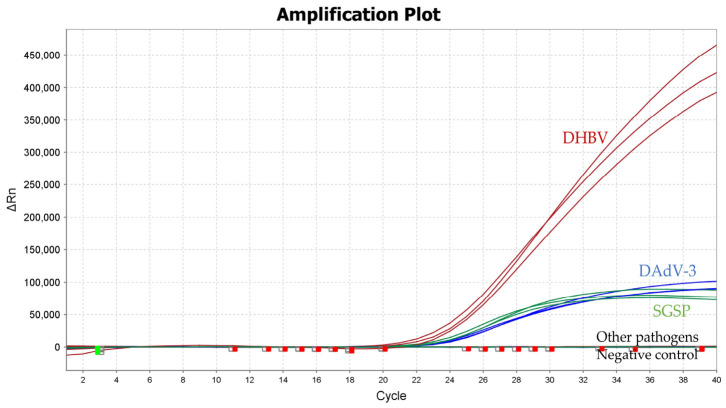
Analytical specific of the triplex qPCR method.

Under the unified optimized reaction conditions, the established triplex qPCR method was used to test nucleic acid templates from common duck pathogens: genomic DNA of DHBV, DAdV-3, SGSP, DPV, MDPV, *E. coli*, Salm, RA, *S. enterica*, *P. multocida*, SGSM, and SGSG, as well as reverse-transcribed cDNA of DHAV-1 and DTMUV. The results verified that DNA extracted from DHBV, DAdV-3 and SGSP yielded distinct fluorescent signals in their corresponding detection channels, whereas no amplification curves were observed for any non-target pathogen templates or negative blank controls. These results fully demonstrate that the developed triplex qPCR assay possesses excellent analytical specificity, and no cross-amplification or cross-reactivity occurs with other prevalent duck pathogens (Figure 4).

### 3.4. Sensitivity Analysis

**Figure 5:**
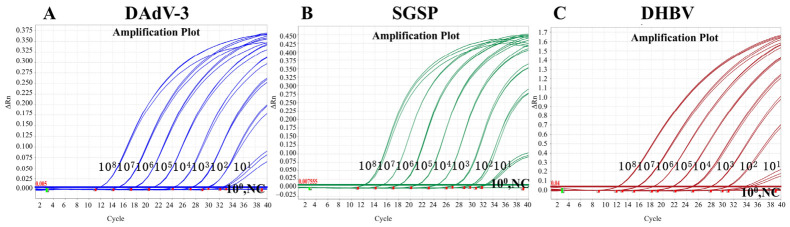
Analytical sensitivity of the triplex qPCR method. Ten-fold serially diluted plasmids (from 10^8^ to 10^0^ copies/µL) were amplified with different primer/probe sets targeting DAdV-3 (**A**), SGSP (**B**), and DHBV (**C**), respectively. NC: negative control.

The results showed that the LOD of the triplex qPCR assay was 10 copies/μL for DAdV-3, SGSP, and DHBV (Figure 5). Table 3 summarizes LOD data from a single experimental batch, with 20 technical replicates performed for each plasmid concentration gradient. At 100 copies/µL, all three target templates displayed 100% positive detection across all replicates. Upon dilution to 10 copies/µL, DHBV and SGSP remained fully detectable, while DAdV-3 exhibited a 95% positive detection rate. At the lowest concentration of 1 copy/µL, detection performance became unstable, with positive rates of 20% (DAdV-3), 35% (SGSP), and 50% (DHBV).

To further validate the reliability of this LOD threshold in accordance with MIQE guidelines, we conducted three independent experimental runs with 20 technical replicates per run for each plasmid concentration; aggregated statistical data from repeated testing are summarized in Supplementary Table S4. Pooled positive rates derived from 60 total replicates per concentration were 98.33% for DAdV-3 and 100% for SGSP and DHBV at 10 copies/μL, with Wilson 95% confidence intervals of 91.14–99.71% and 93.98–100.00%, respectively. As stated in the reference, the standard LOD definition refers to the minimum template concentration that returns positive amplification in ≥95% of replicates [24]. Aligned with this criterion, multi-batch pooled data firmly confirmed that 10 copies/µL serves as the robust LOD for all three target pathogens using this triplex qPCR assay.

### 3.5. Repeatability and Reproducibility of the Triplex qPCR

Serial standard plasmid dilutions at three concentrations (10^7^, 10^5^, and 10^3^ copies/µL) were prepared to assess the intra-assay repeatability and inter-assay reproducibility of the triplex qPCR system. As demonstrated in Table 4, the intra-assay coefficients of variation (CVs) ranged from 0.03% to 1.46%, while inter-assay CVs fell between 0.21% and 1.47%. These low CV values demonstrate that the developed triplex qPCR assay possesses stable repeatability and reproducibility.

### 3.6. Detection of Triplex qPCR Results for Pathogens in Clinical Samples

To assess the clinical applicability of the novel triplex qPCR assay, a total of 215 clinical liver specimens were analyzed. The prevalence rates for DHBV, DAdV-3, and SGSP were 73.95% (159/215), 11.16% (24/215), and 37.21% (80/215), respectively. Co-infection rates were as follows: DHBV/DAdV-3, 5.58% (12/215); DHBV/SGSP, 27.91% (60/215); DAdV-3/SGSP, 2.33% (5/215); and triple infection (DHBV/DAdV-3/SGSP), 2.33% (5/215) (Table 5). In parallel, conventional PCR was performed on the same cohort using established detection criteria for each pathogen. Corresponding detection rates were 67.91% (146/215) for DHBV, 9.30% (20/215) for DAdV-3, and 33.02% (71/215) for SGSP. Co-infection rates by conventional PCR were 4.65% (10/215) for DHBV/DAdV-3, 26.98% (58/215) for DHBV/SGSP, 1.86% (4/215) for DAdV-3/SGSP, and 1.86% (4/215) for triple infection. Overall, the overall concordance between triplex qPCR and conventional PCR was 96.27%, 100%, and 97.67% for DHBV, DAdV-3, and SGSP, respectively. These results collectively demonstrate that the triplex qPCR assay provides enhanced analytical sensitivity, high diagnostic accuracy, and excellent reproducibility compared to conventional PCR, highlighting its robustness for routine diagnostic use.

## 4. Discussion

Duck farming is a vital component of the global livestock industry. However, ducks are highly susceptible to infectious diseases during rearing, leading to substantial economic losses [25,26]. DHBV, DAdV-3, and SGSP are major pathogens responsible for severe liver injury, high mortality, and frequent co-infections in ducks [3,12,15]. Clinically, these pathogens induce overlapping pathological manifestations, including hepatocyte necrosis, hepatic hemorrhage, and pericardial effusion, which complicate rapid and accurate on-site differential diagnosis. Consequently, developing reliable and specific diagnostic assays would be beneficial for identifying the three target pathogens. Traditional diagnostic methods, including virus isolation, bacterial culture, and serological tests, are laborious, time-consuming and insensitive [27]. Although the enzyme-linked immunosorbent assay (ELISA) is widely used, it has limitations such as a narrow quantitative range, potential cross-reactivity, cumbersome operational steps, and a requirement for specific antibodies [28,29]. In contrast to traditional pathogen isolation and identification, quantitative PCR (qPCR) enables rapid, sensitive detection of a broad range of pathogens, making it particularly advantageous for scenarios requiring rapid clinical decision-making [30,31]. With high sensitivity and accuracy, multiplex qPCR allows simultaneous identification of various pathogens in a single reaction, making it a prevalent tool for laboratory testing of poultry infectious agents [32,33]. At present, limited triplex TaqMan qPCR protocols are available for the concurrent screening of DHBV, DAdV-3 and SGSP. In this work, we constructed a triplex TaqMan qPCR assay specific to the three pathogens, providing a complementary diagnostic approach to distinguish infectious agents isolated from clinical duck specimens.

In this study, three pairs of pathogen-specific primers and corresponding TaqMan probes were designed to establish a triplex qPCR assay, enabling simultaneous detection of DHBV, DAdV-3, and SGSP in a single reaction. A multi-target recombinant plasmid, constructed by inserting the DHBV, DAdV-3, and SGSP target gene fragments into a single vector via restriction enzyme sites, was used as the standard plasmid. Compared with three distinct single plasmids, this strategy reduces the expenses of standard plasmid construction and mitigates systematic analytical deviations. The standard curves of the triplex qPCR assay exhibited high linear correlation coefficients, indicating excellent linearity, robust amplification efficiency, and reliable quantitative performance. No cross-reactivity was observed against other prevalent duck pathogens, and both intra-assay and inter-assay coefficients of variation (CVs) were less than 2%, indicating favorable repeatability of this assay. The limit of detection (LOD) of the triplex qPCR for DHBV, DAdV-3, and SGSP was as low as 10 copies/µL. Notably, the LOD of our assay was significantly lower than that of previously reported methods: the real-time qPCR for DAdV-3 established by Wan et al. had an LOD of 40 copies/µL [34], whereas the conventional PCR for DHBV developed by Wang et al. had an LOD of 100 copies/µL [23]. These results confirm that the triplex qPCR assay established in this study is highly practical and exhibits superior sensitivity.

A total of 215 clinical duck liver samples collected from Heilongjiang Province, China, were tested using the triplex qPCR and conventional PCR methods. The positive rates of DHBV, DAdV-3, and SGSP detected by triplex qPCR were 73.95%, 11.16%, and 37.21%, respectively, while those detected by conventional PCR were 67.91%, 9.30%, and 33.02%, respectively. The concordance rates between the two methods were 96.27% for DHBV, 100% for DAdV-3, and 97.67% for SGSP, indicating high reliability of the triplex qPCR assay. Clinical samples frequently presented co-infections involving two or three target pathogens. Such mixed infections can aggravate immunosuppression and inflammatory responses, raise the susceptibility to secondary infection, and lead to more severe disease manifestations [35]. Consistent with this epidemiological feature, our 215 field surveillance samples collected from a limited regional duck farming area contained numerous dual-pathogen co-infected individuals, alongside five specimens that tested positive for all three pathogens simultaneously. These findings indicate a high prevalence of multi-pathogen co-infection within the sampled duck population of this region, rather than representing nationwide epidemiological patterns.

Collectively, these analytical data reveal that the triplex qPCR assay achieves higher analytical sensitivity than conventional single PCR under laboratory conditions. Therefore, this assay serves as a practical molecular tool for rapid identification of DHBV, DAdV-3 and SGSP in duck clinical samples and field epidemiological screening.

This study has several inherent limitations that deserve careful consideration. First, all clinical duck samples were collected from farms within a single province, which restricts the generalizability of our epidemiological observations and the universal applicability of this triplex qPCR assay; further multi-regional field validation covering broader geographical areas is required to confirm its reliability across diverse duck populations. Second, conventional PCR rather than sequencing or bacterial isolation was adopted as the comparative reference method. Conventional PCR is not a recognized gold standard, and only discrepant samples were verified by sequencing while all positive specimens lacked unified gold-standard confirmation, which may bias the consistency analysis between the two detection approaches. Third, the sampling strategy relied mainly on routine surveillance specimens from local farms without a rigorous stratified random sampling design. Accordingly, the high positive rate of DHBV and frequent DHBV/SGSP co-infection observed herein cannot be directly extrapolated to the national duck breeding population. Large-scale systematic epidemiological surveys are necessary to accurately determine the true prevalence of the three pathogens.

## 5. Conclusions

In this study, we established a triplex TaqMan qPCR assay for simultaneous detection of DHBV, DAdV-3 and SGSP. We systematically evaluated its analytical specificity, sensitivity, intra-assay and inter-assay repeatability, and performed parallel detection against conventional single PCR using duck clinical samples collected within this surveillance project. Within the scope of the current sample cohort, this assay shows acceptable analytical performance and enables synchronous screening of the three pathogens. It can serve as an auxiliary molecular testing method to differentiate the three target pathogens for duck samples from the farms enrolled in this study. Further cross-regional field verification was not conducted in the present work, and the applicability of this assay is limited to the sample population investigated herein.

## Figures and Tables

**Table 1:** Primers and probes used in this study.

Name	Sequence (5′-3′)	Product Size (bp)	Reference	GenBank
DAdV-3-F	TACACCTCACAAGCTCATACT	89	This study	KR135164
DAdV-3-R	TGCTCCGCAGCACACT			
DAdV-3-P	VIC-TGACACACTTCAGAAGACACAA-BHQ1			
SGSP-F	CCTGACCGAGCAACGC	83		JN798591
SGSP-R	CCACTCTCACACACGTTCTTCTC			
SGSP-P	CY5-ACAACAGAGCTTTACGATCCGAA-MGB			
DHBV-F	GAGCCCCTWCACCCCAAC	63		MF471769
DHBV-R	ATCTRBCGTGGCTGCTCGAACT			
DHBV-P	FAM-TGCGGGCTCCCCTCTCC-BHQ1			
DAdV-3-F1	ACACTAGACAACGGAGGCCT	548	Fujian provincial local standard of China DB35/T 1872-2019 [21]	-
DAdV-3-R1	AATCTTGATACGTAATCATACACA			
DHBV-F1	GGACTCGAACCTAGAAGAA	159	Wang et al., 2013 [22]	-
DHBV-R1	TTTATTTCCTAGGCGAGGG			
SGSP-F1	GAGTTTGATCMTGGCTCAG	750	Wilson et al., 1990 [23]	-
SGSP-R1	CTAHAGGGTATCTAATCCT			

**Table 2:** Triplex qPCR reaction mixture.

Component	Volume (µL)
Premix Ex Taq (Probe qPCR) (2×)	10
DAdV-3-F (10 µM)	0.7
DAdV-3-R (10 µM)	0.7
DAdV-3-P (10 µM)	0.5
SGSP-F (10 µM)	0.8
SGSP-R (10 µM)	0.8
SGSP-P (10 µM)	0.6
DHBV-F (10 µM)	0.9
DHBV-R (10 µM)	0.9
DHBV-P (10 µM)	0.7
ROX Reference Dye	0.2
ddH_2_O	1.2
Template	2
Total volume	UP to 20

**Table 3:** Positive detection rates of serially diluted standard plasmids (100, 10, and 1 copy/µL) across 20 replicate tests.

Pathogen	Concentration	Positive Number	Positive Detection Rate	95% CI of Positive Rate (Wilson)
DAdV-3	100 copies/μL	20	100%	83.89–100.00%
	10 copies/μL	19	95%	76.39–99.11%
	1 copy/μL	4	20%	8.07–41.60%
SGSP	100 copies/μL	20	100%	83.89–100.00%
	10 copies/μL	20	100%	83.89–100.00%
	1 copy/μL	7	35%	18.12–56.71%
DHBV	100 copies/μL	20	100%	83.89–100.00%
	10 copies/μL	20	100%	83.89–100.00%
	1 copy/μL	10	50%	29.93–70.07%

**Table 4:** Repeatability of the triplex qPCR.

		Intra-Assay			Inter-Assay		
Pathogen	Templates (Copies/µL)	Mean Ct Value	SD	CV (%)	Mean Ct Value	SD	CV (%)
DAdV-3	10^7^	15.34	0.22	1.46	15.48	0.23	1.47
	10^5^	21.18	0.15	0.69	21.37	0.20	0.96
	10^3^	27.47	0.02	0.09	27.70	0.18	0.64
SGSP	10^7^	15.45	0.17	1.11	15.37	0.12	0.80
	10^5^	21.33	0.05	0.23	21.30	0.04	0.21
	10^3^	27.54	0.01	0.03	27.54	0.22	0.79
DHBV	10^7^	15.41	0.15	0.97	15.26	0.20	1.31
	10^5^	21.36	0.03	0.13	21.24	0.12	0.58
	10^3^	27.78	0.08	0.28	27.62	0.30	1.10

**Table 5:** Comparison between the triplex qPCR method and conventional PCR method with clinical samples.

Pathogens		Conventional PCR+	Conventional PCR−	Total
DHBV	Triplex qPCR **+**	74	8	82
	Triplex qPCR **−**	0	133	133
	total	74	141	215
	Compliance rate = (74 + 133)/215 = 96.27%			
DAdV-3		**Conventional PCR** **+**	**Conventional PCR** **−**	**total**
	Triplex qPCR **+**	2	0	2
	Triplex qPCR **−**	0	213	213
	total	2	213	215
	Compliance rate = (2 + 213)/215 = 100%			
SGSP		**Conventional PCR** **+**	**Conventional PCR** **−**	**total**
	Triplex qPCR **+**	5	5	10
	Triplex qPCR **−**	0	205	205
	total	5	210	215
	Compliance rate = (5 + 205)/215 = 97.67%			
DHBV + DAdV-3		**Conventional PCR** **+**	**Conventional PCR** **−**	**total**
	Triplex qPCR **+**	10	2	12
	Triplex qPCR **−**	0	203	203
	total	10	205	215
	Compliance rate = (10 + 203)/215 = 99.07%			
DHBV + SGSP		**Conventional PCR** **+**	**Conventional PCR** **−**	**total**
	Triplex qPCR **+**	58	2	60
	Triplex qPCR **−**	0	155	155
	total	58	157	215
	Compliance rate = (58 + 155)/215 = 99.07%			
DAdV-3 + SGSP		**Conventional PCR** **+**	**Conventional PCR** **−**	**total**
	Triplex qPCR **+**	4	1	5
	Triplex qPCR **−**	0	210	210
	total	4	211	215
	Compliance rate = (4 + 210)/215 = 99.53%			
DHBV + DAdV-3 + SGSP		**Conventional PCR** **+**	**Conventional PCR** **−**	**total**
	Triplex qPCR **+**	4	1	5
	Triplex qPCR **−**	0	210	210
	total	4	211	215
	Compliance rate = (4 + 210)/215 = 99.53%			

## Data Availability

The original contributions presented in this study are included in the article/Supplementary Material. Further inquiries can be directed to the corresponding author(s).

## Supplementary Materials

The following supporting information can be downloaded at: https://www.mdpi.com/article/10.3390/vetsci13070692/s1, Table S1: Sequences of three tandem target segments in the fusion standard plasmid Fusion-Std-SGSP-DAdV3-DHBV; Table S2: Reaction mixtures and thermal cycling protocols of three PCR assays; Table S3. Maximum ΔRn (end-point fluorescence intensity) and average Ct values of three target genes under different annealing temperatures. Table S4. Limit of detection (LOD) validation data based on three independent amplification runs for DHBV, DAdV-3, and SGSP; Figure S1. Amplification curves of three target genes at annealing temperatures of 54 °C, 56 °C, 58 °C and 60 °C. (**A**) DAdV-3; (**B**) SGSP; (**C**) DHBV.
