# Supplementary material for: Development of a Triplex TaqMan Real-Time PCR Assay for Simultaneous Detection of Duck Hepatitis B Virus, Duck Adenovirus Type 3, and Streptococcus gallolyticus subsp. pasteurianus
Source: Vet Sci. 2026 Jul 15;13(7):692. doi: 10.3390/vetsci13070692 (PMC13431575; doi:10.3390/vetsci13070692)
Supplement: Supplementary file 1 [file vetsci-13-00692-s001.zip › vetsci-4371737-supplementary.pdf]

**Table S1.** Sequences of three tandem target fragments within fusion standard plasmid Fusion-Std-SGSP-DAdV-3-DHBV.

| **Fusion-Std-SGSP-DAdV3-DHBV** | **Sequence (5' - 3')** | **Size (bp)** |
|---|---|---|
| SGSP 16S rRNA segment | CCGACCTGAGAGGGTGATCGGCCACACTGGGACTGAGACACGGCCCAGACTCCTACGGGAGG<br>CAGCAGTAGGGAATCTTCGGCAATGGGGGCAACCCTGACCGAGCAACGCCGCGTGAGTGAAG<br>AAGGTTTTCGGATCGTAAAGCTCTGTTGTAAGAGAAGAACGTGTGTGAGAGTGGAAAGTTCAC<br>ACAGTGACGGTAACTTACCAGAAAGGGACGGCTAACTACGTGCCAGCAGCCGCGGTAATACG<br>TAGGTCCCGAGCGTTGTCCGGATTTATTGGGCGTAAAGCGAGCGCAGGCGGTTTAATAAGTCT<br>GAAG | 316 |
| DAdV-3 segment | TCACTGACAATACCCTACACCTCACAAGCTCATACTCAACCTATGTCTTTACGAGTGGATCTGA<br>CACACTTCAGAAGACACAAGCCCAAGTGTGCTGCGGAGCAGGGTCTGTTACGTTTCCGTGCGC<br>ATACTATGCCAAGATCGTATGCTCAAACAATGTGTCTTCAGGATATATAACACTGAAGGTGAG<br>CGCTGAGGATGCATCACATGCTGTAGATCAACGCTTCGCGACTATTCAACCAGTATTCACATTC | 359 |

| | | |
|---|---|---|
| | TGGTTATGTCGAGACATAGGTAATGAAAACACCGTCAATTTTTCCCACTGTACCAACAACAGTT<br>ATAAGCCAGAGGAAACCGCAGTCGTTAAGGCATGCATCACA | |
| DHBV segment | GCCCCGACCATTGAAGCAATCACTAGACCAATCCAAGTGGCTCAGGGAGGCAGAAAAACAAC<br>TACGGGTACTAGAAAACCTCGTGGACTCGAACCTAGAAGAAGAAAAGTTAAAACCACAGTTG<br>TCTATGGGAGAAGACGTTCAAAGTCCCGGGAAAGGAGAGCCCCTACACCCCAACGTGCGGGC<br>TCCCCTCTCCCACGTAGTTCGAGCAGCCACCATAGATCTCCCTCGCCTAGGAAATAAATTACCT<br>GCTAGGCATCACTTAGGTAAATTGTCAGGACTATATCAAATGAAGGGCTGTACTTTTAACCCA<br>GAATGGAAAGTACCAGATATTTCGGATACTCATTTT | 349 |

**Table S2** Reaction mixtures and thermal cycling protocols of three PCR assays.

| Standard | Pathogen | Method | Reagent | Volume (μL) | Protocol |
|---|---|---|---|---|---|
| | | | 10×PCR buffer | 2.5 μl | |
| | | | DAdV-3-F(20μM) | 1 μL | pre-denaturation at 94°C for 5 min |
| | | | DAdV-3-R(20μM) | 1 μL | followed by 35 cycles contain: |
| | | | dNTP Mixture | 2 μL | denaturation at 94°C for 50 s |
| DB35/T 1872-2019 | DAdV-3 | PCR | *Taq* polymerase | 0.5 μL | annealing at 55°C for 30 s |
| | | | nuclease-free $H_2O$ | 16 μL | extension at 72°C for 35 s |
| | | | Template | 2 μL | final extension at 72°C for 10 min |
| | | | Total volume | 25 μL | |
| | | | 2×Taq Master Mix | 12.5 μL | pre-denaturation at 94°C for 5 min |
| The PCR (Wilson et al, | | | SGSP-F(10μM) | 1 μL | followed by 35 cycles contain: |
| 1990) reaction mixture | SGSP | PCR | SGSP-R(10μM) | 1 μL | denaturation at 94°C for 50 s |

| | | | | | |
|---|---|---|---|---|---|
| and protocol | | | nuclease-free $H_2O$ | 8.5 μL | annealing at 55°C for 30 s |
| | | | Template | 2 μL | extension at 72°C for 1 min |
| | | | Total volume | 25 μL | final extension at 72°C for 3 min |
| The PCR (Wang et al, 2013) reaction mixture and protocol | DHBV | PCR | 10×PCR buffer | 2.5 μL | |
| | | | DHBV-F(20μM) | 1 μL | pre-denaturation at 94°C for 2 min |
| | | | DHBV-R(20μM) | 1 μL | followed by 40 cycles contain: |
| | | | dNTP Mixture | 0.5 μL | denaturation at 94°C for 15 s |
| | | | *Taq* polymerase | 0.5 μL | annealing at 49°C for 30 s |
| | | | nuclease-free $H_2O$ | 17.5 μL | extension at 72°C for 30 s |
| | | | Template | 2 μL | |
| | | | Total volume | 25 μL | |

**Table S3.** Maximum ΔRn (end-point fluorescence intensity) and average Ct values of three target genes under different annealing temperatures.

| Annealing temperature | Pathogen | Mean Ct value | Maximum ΔRn（End-point fluorescence intensity） | Amplification performance evaluation |
|---|---|---|---|---|
| 54℃ | DAdV-3 | 16.833 | 2.175 | Lower fluorescence intensity and delayed Ct values |
| 54℃ | SGSP | 17.213 | 1.549 | |
| 54℃ | DHBV | 17.416 | 6.656 | |
| 56℃ | DAdV-3 | 15.824 | 2.419 | Highest fluorescence peak, minimal Ct value, and no non-specific amplification peaks. |
| 56℃ | SGSP | 16.296 | 1.703 | |
| 56℃ | DHBV | 16.162 | 7.621 | |
| 58℃ | DAdV-3 | 17.047 | 2.354 | Lower fluorescence intensity and delayed Ct values |
| 58℃ | SGSP | 17.557 | 1.696 | |
| 58℃ | DHBV | 17.294 | 6.968 | |
| 60℃ | DAdV-3 | 16.695 | 1.987 | Lower fluorescence intensity and |

| | | | | |
|---|---|---|---|---|
| 60℃ | SGSP | 17.094 | 1.579 | delayed Ct values |
| 60℃ | DHBV | 16.635 | 6.081 | |

**Table S4.** Limit of detection (LOD) validation data based on three independent amplification runs for DHBV, DAdV-3, and SGSP.

| Pathogen | Concentration | Independent run 1 (Positive/20) | Independent run 2 (Positive/20) | Independent run 3 (Positive/20) | Combined positive rate | Positive detection rate(%) | 95% CI of positive rate (Wilson)(%) |
|---|---|---|---|---|---|---|---|
| DAdV-3 | 100 copies/μL | 20 | 20 | 20 | 60/60 | 100 | 93.98–100.00 |
| | 10 copies/μL | 19 | 20 | 20 | 59/60 | 98.33 | 91.14–99.71 |
| | 1 copy/μL | 4 | 5 | 3 | 12/60 | 20 | 11.83–31.78 |

| | | | | | | | |
|---|---|---|---|---|---|---|---|
| | 100 copies/μL | 20 | 20 | 20 | 60/60 | 100 | 93.98–100.00 |
| SGSP | 10 copies/μL | 20 | 20 | 20 | 60/60 | 100 | 93.98–100.00 |
| | 1 copy/μL | 7 | 6 | 4 | 17/60 | 28.33 | 18.51–40.77 |
| | 100 copies/μL | 20 | 20 | 20 | 60/60 | 100 | 93.98–100.00 |
| DHBV | 10 copies/μL | 20 | 20 | 20 | 60/60 | 100 | 93.98–100.00 |
| | 1 copy/μL | 10 | 8 | 7 | 25/60 | 41.67 | 30.06–54.27 |

[a]Combined positive rate was calculated as the total number of positive replicates across three independent runs divided by the total 60 replicates (20 technical replicates per run). The 95% confidence interval of positive detection rate was computed via Wilson score interval with z = 1.96 for 95% confidence level, which is appropriate for binomial proportion data including extreme detection rates of 100%.

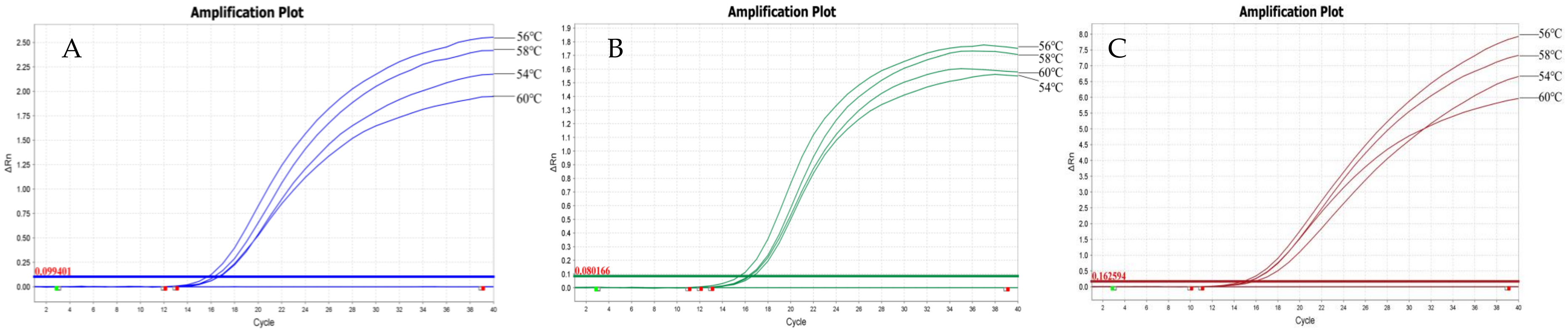


Figure S1. Amplification curves of three target genes at annealing temperatures of 54 ℃, 56 ℃, 58 ℃ and 60 ℃. (A) DAdV-3; (B) SGSP; (C) DHBV.
